# National contributions to climate change due to historical emissions of carbon dioxide, methane, and nitrous oxide since 1850

**DOI:** 10.1038/s41597-023-02041-1

**Published:** 2023-03-29

**Authors:** Matthew W. Jones, Glen P. Peters, Thomas Gasser, Robbie M. Andrew, Clemens Schwingshackl, Johannes Gütschow, Richard A. Houghton, Pierre Friedlingstein, Julia Pongratz, Corinne Le Quéré

**Affiliations:** 1 Tyndall Centre for Climate Change Research, School of Environmental Sciences, University of East Anglia (UEA), Norwich, UK; 2 CICERO Center for International Climate Research, Oslo, Norway; 3 International Institute for Applied Systems Analysis (IIASA), Laxenburg, Austria; 4 Ludwig Maximilian University of Munich, Munich, Germany; 5 Department of Transformation Pathways, Potsdam Institute for Climate Impact Research, Potsdam, Germany; 6 Woodwell Climate Research Center, Falmouth, MA USA; 7 College of Engineering, Mathematics and Physical Sciences, University of Exeter, Exeter, UK; 8 Max Planck Institute for Meteorology, Hamburg, Germany

**Keywords:** Climate change, Energy and society, Environmental impact

## Abstract

Anthropogenic emissions of carbon dioxide (CO_2_), methane (CH_4_) and nitrous oxide (N_2_O) have made significant contributions to global warming since the pre-industrial period and are therefore targeted in international climate policy. There is substantial interest in tracking and apportioning national contributions to climate change and informing equitable commitments to decarbonisation. Here, we introduce a new dataset of national contributions to global warming caused by historical emissions of carbon dioxide, methane, and nitrous oxide during the years 1851–2021, which are consistent with the latest findings of the IPCC. We calculate the global mean surface temperature response to historical emissions of the three gases, including recent refinements which account for the short atmospheric lifetime of CH_4_. We report national contributions to global warming resulting from emissions of each gas, including a disaggregation to fossil and land use sectors. This dataset will be updated annually as national emissions datasets are updated.

## Background and Summary

Anthropogenic emissions of carbon dioxide (CO_2_), methane (CH_4_) and nitrous oxide (N_2_O) are key components responsible for climate change since the pre-industrial period^1,2^. The use of fossil carbon sources in the energy, industry, transport, waste and product use sectors and land use, land use change and forestry (LULUCF), has led to increased atmospheric concentrations of CO_2_, CH_4_ and N_2_O and driven Earth’s surface energy balance into surplus^3–6^. The Intergovernmental Panel on Climate Change (IPCC) estimated in its Sixth Assessment Report (AR6) that rising atmospheric concentrations of CO_2_, CH_4_ and N_2_O have already caused global mean surface temperature (GMST) to increase by 1.4 °C (0.9–2.2 °C at the 90% confidence interval) in the industrial era, independent of other greenhouse gases (GHGs), ozone precursors (e.g., VOC, CO, NO_x_), and aerosols (e.g., SO_2_, black carbon and organic carbon) which either heat or cool the planet for a net warming of 1.1 °C attributable to human activities^1,2,7,8^.

Due to their long-lived or powerful effects on climate, the national emissions of CO_2_, CH_4_ and N_2_O are widely regulated by the United Nations Framework Convention on Climate Change (UNFCCC). All parties to the convention set targets for CO_2_ in the form of nationally determined contributions (NDCs)^9^, while around 90% of NDCs include targets for CH_4_ and N_2_O. Consequently, keeping track of the emissions of CO_2_, CH_4_ and N_2_O and the climatic responses to those emissions is especially important for ensuring accountability with respect to NDCs. The current work seeks to inform the 2023 Global Stocktake of the UNFCCC, the formal process by which national progress on NDCs is evaluated. While we focus on three CO_2_, CH_4_ and N_2_O that are included in most NDCs, we note that future work should seek to include other important GHGs, such as fluorinated gases (F-gases), which are also included in the NDCs of some countries.

The contributions of individual countries to the warming caused by rising CO_2_, CH_4_ and N_2_O concentrations has changed through time and depends strongly on the unique history of land clearing, industrialisation, and decarbonisation of a country. Emissions of fossil CO_2_ – the largest driver to historical climate change^1^ – have generally continued to rise with economic growth in developing countries even after the establishment of the UNFCCC in 1992. On the other hand, fossil CO_2_ emissions in developed countries have begun to decline after increasing throughout much of the industrial era since the mid-19^th^ Century^3,10,11^. National contributions to climate change are closely tied to cumulative emissions of CO_2_ in the industrial era because a substantial fraction of emitted CO_2_ remains in the Earth’s atmosphere for centuries^6,12^. Consequently, emissions from developed nations have contributed significantly to warming since the industrial revolution^12,13^. Tracking national contributions to climate change is thus critical to understanding the burden of responsibility that a country carries for global warming and can further inform the design of international policies that pursue equitable decarbonisation pathways.

Numerous studies have assessed national contributions to the historical change in GMST caused by anthropogenic emissions of CO_2_ and other gases or aerosols. These studies have typically employed simple climate models to simulate the GMST response to the emissions of CO_2_^14–17^, CH_4_ and N_2_O^18–20^, and other gases and aerosols^12,13,21–26^. Several studies have further evaluated how national contributions to warming may develop in future periods based on current pledges, nationally declared contributions or emission scenarios^17,25,27^. Others have employed coupled climate models to evaluate national contributions to simulated climate change impacts resulting from warming, such as the likelihood of extreme weather, sea level rise, and economic growth^17,20,28,29^. Within these studies, each climate model or ensemble of models exhibits a specific climate sensitivity such that the response of GMST to anthropogenic emissions is model- or ensemble-specific^30^.

Recent advances in the understanding of the response of GMST to cumulative CO_2_ emissions have provided express formulas that can be used to efficiently calculate the warming resulting from cumulative emissions of CO_2_^6,31–34^. The transient climate response to cumulative emissions of CO_2_ (TCRE), a coefficient representing the warming per trillion tonnes of carbon emitted as CO_2_, was estimated in the IPCC’s Sixth Assessment Report to be 0.45 °C per 10^3^ Pg CO_2_ (0.3–0.6 °C per 10^3^ Pg C at the 90% confidence interval) based on synthesis of estimates made in 28 earlier studies (see **Methods**; Eq. (1)). In addition, global warming potential (GWP) metrics have been developed to relate emissions of non-CO_2_ greenhouse gas emissions to cumulative CO_2_ emissions, most recently including the GWP* approach which captures the contrasting impacts of short and long -lived climate pollutants on GMST^35–39^. An advantage of using express formulas based on TCRE and GWP* coefficients to calculate the warming response to emissions is that the values of each coefficient can be selected in line with IPCC best-estimates and uncertainty ranges, which synthesise the behaviour shown by many different models across dozens of studies^2,6^. Hence, the climatic response to emissions of GHGs does not depend on the climate sensitivity of an individual model. Other straightforward equations (see **Methods**; Eqs. (2, 3)) have been devised to relate the warming effect of CH_4_ and N_2_O emissions to the warming effect of cumulative CO_2_ emissions over defined time horizons, often 100 years^36–38^. The express equations are yet to be applied to estimate the combined effects of the national emissions of multiple gases on GMST.

Here, we present a new dataset of changes in GMST during 1851–2021 resulting from historical emissions of CO_2_, CH_4_ and N_2_O at the global scale and for individual countries. Our estimates of warming are based on the application of express equations for TCRE and Global Warming Potential (GWP) to the emissions time series for each gas, using current best-estimates of the coefficients involved in the equations from IPCC AR6 (see **Methods**)^3,40–43^. We further estimate the contributions of fossil and LULUCF emissions to change in GMST at global and national levels. To enhance the relevance of the dataset to international organisations, we also provide estimates of the contributions of various country groupings including Annex I countries (number of countries, n = 42), Annex II countries (n = 23), economies in transition (EITs; n = 15), the least developed countries (LDCs; n = 47), and like-minded developing countries (LMDC; n = 24) as defined by the UNFCCC. We also consider the contributions of the organisation for economic co-operation and development (OECD; n = 38), the European Union (EU27 post-Brexit), and the Brazil, South Africa, India and China (BASIC) group. Lists of the countries included in each country grouping are provided with the **Data Records**^44^.

This dataset is publicly available via a Zenodo repository^44^. For completeness, we provide the annual and cumulative emissions data used to calculate the change in GMST. We emphasise that the emissions data are not introduced here but rather derive from the Global Carbon Project (GCP)^3,40^ and the national historical emissions time series component of the Potsdam Realtime Integrated Model for probabilistic Assessment of emissions Paths dataset (PRIMAP-hist)^41,42^. We focus on the period since 1850 to align with the period over which warming was usually evaluated in the IPCC AR6^45^. The final year of 2021 is determined by the mutual period covered by the requisite datasets. We will update our dataset annually in line with updates and extensions to the GCP^3,40^ and PRIMAP-hist^41,42^ emissions datasets.

## Methods

### Emissions time series

#### Carbon dioxide

We retrieved estimates of territorial fossil CO_2_ emissions for the years 1850–2021 from the GCP, as published in its 2021 assessment of the global carbon budget (GCB)^3,40^. The GCB estimates include national annual emissions of CO_2_ due to coal, oil and natural gas combustion, the use of these fuels in non-combustive industrial processes, and the production of cement clinkers^3,40^. The emissions relate to the energy, industry, transport, product use, solvent use and waste sectors. Under the GCB methodology, national CO_2_ emissions are preferentially taken from the country submissions to the United Nations Framework Convention on Climate Change (UNFCCC) for 42 Annex I countries. Emissions in other countries and in Annex I countries prior to 1990 predominantly derive from the Carbon Dioxide Information Analysis Center (CDIAC)^46^. For the years since 2020, not included in either the UNFCCC or CDIAC datasets, the national emissions are estimated using national or regional energy growth rates from the annual BP Statistical Review of World Energy^47^. Cement emissions are based on national inventories of cement production and ratios of clinker production from officially reported clinker production data and emission factors, IPCC default emission factors, industry-reported clinker production, and survey-based clinker ratios^48^. A more complete description of methodology used to compile the fossil CO_2_ emissions time series is provided in our previous work (refs. ^3,40^).

In addition, we retrieved estimates of historical CO_2_ emissions from LULUCF for the years 1850–2021 from the average of the three bookkeeping models that contribute to the GCB (BLUE, Houghton & Nassikas [H&N], and OSCAR)^49–51^. The bookkeeping models combine historical estimates of changes in agricultural and forest areas, other loss of primary vegetation and wood harvesting with response curves that describe the decay of vegetation and soil carbon over time, including transfer to product pools of different lifetimes, as well as carbon uptake due to regrowth. Emissions from peat burning and drainage are added from external datasets^52–55^. This way gross emissions and removals due to deforestation, afforestation, logging and forest degradation (including harvest activity), shifting cultivation (cycle of cutting forest for agriculture, then abandoning), conversion of pastures and grasslands, and regrowth of forests following wood harvest or abandonment of agriculture are captured^3^. A key difference between the bookkeeping estimates is their source of historical land-use data: gridded data from the Land Use Harmonization dataset (LUH2)^56^ in the case of BLUE; national data from the Forest Resource Assessment (FRA)^57^ and agricultural areas^58^ from the United Nations Food and Agriculture Organisation (FAO) in H&N, and; a combination of LUH2 and FRA data in OSCAR). All datasets are ultimately based on information of FAO agricultural area and national wood harvest statistics, both available since 1961, or forest area, available since 1990. The information is extended to earlier time periods using other sources of information such as population growth or regional historical sources^50,59^. While the minimum spatial units in H&N and OSCAR are countries or country groupings, simulations with BLUE are spatially explicit at quarter-degree resolution and carbon fluxes aggregated afterwards to the country level.

Using the abovementioned datasets, we constructed a time series of cumulative total, fossil and LULUCF CO_2_ emissions for the years 1851–2021 (i.e. since the base year of 1850). Estimates are provided for each country and country grouping as well as for the global total.

#### Methane and nitrous oxide

We retrieved estimates of historical N_2_O emissions for the years 1850–2021 and CH_4_ emissions for the years 1830–2021 from the Potsdam Realtime Integrated Model for probabilistic Assessment of emissions Paths dataset (PRIMAP-hist version 2.4)^41,42^. PRIMAP-hist combines various datasets into a global multi-gas emissions record. For CH_4_, the data for years 1830–1849 are required to estimate warming for the years 1850 through 1869 (see ***Non-CO***_***2***_ ***Short-lived Climate Forcers***, below).

For CH_4_ and N_2_O, the fossil emissions estimates in PRIMAP-hist relate to emissions from energy, industry, transport, product use, solvent use and waste. LULUCF emissions in PRIMAP-hist HISTTP include emissions caused by land use changes, such as deforestation or agricultural land abandonment, and agricultural emissions of N_2_O and CH_4_ including various sources, such as rice cultivation, synthetic fertilizers, and manure management (sectors ‘livestock’ and ‘agriculture excluding livestock’). PRIMAP-hist adopts data from various underlying datasets from the UNFCCC^60^, the Food and Agriculture Organization (FAO)^42,58^, the Emissions Database for Global Atmospheric Research (EDGAR)^61,62^, and the Community Emissions Data System (CEDS)^63^. Details of the prioritisation given to the various data sources included in PRIMAP-hist are described in ref. ^41^. We selected the HISTTP scenario of the PRIMAP-hist dataset, in which emissions estimates based on third-party datasets (e.g. research institutes, international organisations, or private companies) are prioritized over country-reported data, rather than the HISTCR scenario in which country-reported data are prioritised over third-party datasets. This selection is made because the estimates of the PRIMAP-hist HISTTP scenario are closer to the average of a wider range of emissions products than the HISTCR scenario during the period 1970–2018^64^.

Using the abovementioned datasets, we constructed a time series of cumulative total, fossil and LULUCF emissions of CH_4_ and N_2_O for each year during 1851–2021 (since the base year 1850), all expressed in CO_2_-equivalent terms (see section ‘**CO**_**2**_**-equivalent Emissions of Non-CO**_**2**_ **Species**’). Estimates are provided for each country and country grouping as well as for the global total.

### Global mean surface temperature response to cumulative co_2_ emissions

In recent years, methods for the express calculation of the GMST responses to cumulative emissions of CO_2_ and non-CO_2_ gases have emerged. A range of studies demonstrated that the CO_2_-induced GMST increase is proportional to cumulative carbon emissions of CO_2_, regardless of the path taken to reach peak cumulative CO_2_ emissions^6,31–34^. Consequently, a constant known as the transient climate response to cumulative emissions of CO_2_ (TCRE) can be defined as the change in GMST per 1,000 Pg C emitted (°C per 10^3^ Pg C). In its AR6^6^, the IPCC synthesised estimates of TCRE from dozens of model and observation-based studies of TCRE and arrived at the current best estimate of 1.65 °C per 10^3^ Pg C emitted (likely range 1.0–2.3 °C per 10^3^ Pg C).

The GMST response to cumulative emissions of CO_2_ can be expressly calculated as follows:

1 $$\Delta T=\kappa \times \frac{1}{C}\times {E}_{C{O}_{2}}$$

where Δ*T* is the change in GMST (°C), *k* is the TCRE (°C per 10^3^ Pg CO_2_ emitted), ${E}_{C{O}_{2}}$is the cumulative emissions of CO_2_ during the period of interest (10^3^ Pg CO_2_), and *C* is a constant used to convert the mass of carbon in a CO_2_ molecule to the total mass of a CO_2_ molecule (*C* = 3.664 Pg CO_2_ Pg C^−1^)^3^. Note that the IPCC’s central estimate for TCRE in CO_2_ terms $(\kappa \times \frac{1}{C})$ is 0.45 °C per 10^3^ Pg CO_2_ emitted.

The TCRE has previously been used to make express calculations of the cumulative emissions of CO_2_ that remain to be emitted before GMST exceeds a chosen warming target (e.g. 1.5 °C or 2.0 °C), known as the remaining carbon budget^65–68^. For example, the remaining carbon budget is regularly re-assessed in the United Nations Environment Programme’s Gap Report^69^. It has also been used in previous work to expressly calculate national contributions to warming caused by CO_2_ emission^12^.

Here, we use Eq. (1) to estimate the GMST response to cumulative CO_2_ emissions in years 1851–2021 (since the base year 1850), adopting the IPCC best-estimate for TCRE (0.45 °C per 10^3^ Pg CO_2_).

### CO_2_-equivalent emissions of Non-CO_2_ species

#### Non-CO_2_ long-lived climate forcers (N_2_O)

The GMST response to emissions of other long-lived climate forcers (LLCFs) can be estimated by first expressing their emissions in terms of the cumulative CO_2_ emissions that would result in equivalent warming over a selected time horizon. For conversion of cumulative non-CO_2_ LLCF emissions to an equivalent quantity of cumulative CO_2_ emissions, one widely used metric is the global warming potential (GWP)^2^. GWP expresses the time-integrated radiative forcing (warming) caused by a pulse emission of a non-CO_2_ greenhouse gas, relative to a pulse emission of an equal mass of CO_2_ (refs. ^70,71^). The GWP of a gas depends on its radiative efficiency (infrared energy absorbed) relative to that of CO_2_, which has been determined using spectroscopy across a range of gas mixing ratios relevant to the study of Earth’s climate^70^. In addition, the GWP of a gas depends on its atmospheric lifetime and the time horizon of interest. As the residence time of a non-CO_2_ GHG in the atmosphere can be longer (or shorter) than that of CO_2_, the proportion of the non-CO_2_ gas that remains in the atmosphere at any time following the pulse emission can be greater (or smaller) than the proportion of CO_2_ that remains in the atmosphere and the original pulse emission can thus have a more (or less) lasting impact on the energy balance of the atmosphere. Consequently, the GWP metric is dependent on time since emission; its value at a relatively near time horizon (e.g. 20 years) can differ from its value at a later time horizon (e.g. 100 years).

GWP values calculated over a time horizon of 100 years (denoted GWP_100_) have been employed particularly extensively in climate studies to report emissions of various GHGs on the same scale as CO_2_ (ref. ^2^). GWP_100_ values are used to estimate cumulative CO_2_-equivalent emissions of an LLCF over a 100-year time horizon, denoted ${E}_{C{O}_{2}-{e}_{100}}$ (Pg CO_2_-e_100_) in Eq. (2):﻿

2 $${E}_{C{O}_{2}-{e}_{100}}={E}_{LL}\times {GWP}_{100}$$

where E_LL_ is the cumulative emissions of the LLCF (Pg LLCF) and GWP_100_ is a constant representing the mass of CO_2_ that would result in equivalent warming over a 100-year time horizon (unitless). For example, the current best estimate for the GWP_100_ of N_2_O is 273 (ref. ^2^), signifying that 1 Pg of N_2_O results in the same warming over a 100-year horizon as 273 Pg of CO_2_.

Here, we use Eq. (2) to estimate the cumulative CO_2_-equivalent (${E}_{C{O}_{2}-{e}_{100}}$) emissions of N_2_O (an LLCF) for years 1851–2021, adopting a GWP_100_ value for N_2_O of 273 as reported in IPCC AR6^2,39^. We substituted the result (${E}_{C{O}_{2}-{e}_{100}}$) into Eq. (1) in place of ${E}_{C{O}_{2}}$ to estimate the GMST response to emissions of N_2_O during 1851–2021 (since the base year 1850). We adopted the TCRE value of 0.45 °C per 10^3^ Pg CO_2_ in Eq. (1), as discussed above (see section ‘**Global Mean Surface Temperature Response to Cumulative CO**_**2**_ **Emissions**’).

#### Non-CO_2_ short-lived climate forcers (CH_4_)

Recent studies have highlighted that the nature of the GMST response to emissions of short-lived climate forcers (SLCFs), including CH_4_, differs considerably from the GMST response to LLCFs^39,72,73^. Due to the short atmospheric lifetime of SLCFs (e.g. ~9 years for CH_4_), the atmospheric concentration of a SLCF re-equilibrates within a short period following an increase or decrease in annual emissions. Consequently, the radiative forcing also stabilises well within a 100-year time horizon and the GMST response to SLCF emissions is not simply proportional to cumulative emissions as in the case of LLCFs. Rather, the GMST response to historical CH_4_ depends foremost on recent changes in the rate of annual emissions and to a lesser extent on cumulative longer-term emissions^36,38,39^.

To account for the differing dynamics of SLCFs in the atmosphere over long time horizons, recent work has focussed on providing an adaptation to Eq. (2) that includes the response of GMST to both cumulative SLCF emissions and recent changes in the rate of SLCF emissions^35–39^. The resulting method, referred to as the GWP* approach, calculates the cumulative CO_2_-equivalent emissions over a 100-year time horizon (${E}_{C{O}_{2}-{e}_{100}}$, unit Pg CO_2_−e_100_) of an SLCF using Eq. (3):

3 $${E}_{C{O}_{2}-{e}_{100}}=sg({E}_{SL}\times {GWP}_{100})+(1-s)g\left(\frac{\Delta {E}_{SL(t-\Delta t)}\times {GWP}_{100}\times H}{\Delta t}\right)$$

where $\Delta {E}_{SL(t-\Delta t)}$ is the change in cumulative emissions (Pg SLCF) between year t-Δ*t* and year *t*, with Δ*t* representing a recent time period (years) during which cumulative SLCF emissions have evolved (e.g. 20 years). Note that $\frac{\Delta {E}_{SL\left(t-\Delta t\right)}}{\Delta t}$ is equivalent to the net change in the annual rate of SLCF emissions during the period Δ*t*. *H* (years) is the time horizon of interest consistent with the time horizon of the GWP metric (e.g. 100 years is used for the GWP_100_ metric). Previous studies have demonstrated the validity of Eq. (3) with Δ*t* = 20 years and H = 100 (refs. ^36,38^) and thus we also adopt these values in the current work.

The coefficient *s* shares a relationship with the rate of decline of radiative forcing resulting from the deep ocean thermal adjustment to recent forcing changes, *ρ* $\left(\rho =\frac{s}{H(1-s)}\right)$ (ref. ^36^). Based on the optimised reproduction of the GMST response given by climate models across multiple emission scenarios, *s* = 0.25 for *H* = 100 and *ρ* = 0.33% year^−1^ (ref. ^36^). The value of *ρ* = 0.33% year^−1^ derives from a standard IPCC impulse-response model^36,74,75^. Note that the small value of *s* indicates that the impact of SLCF emissions on GMST is primarily determined by recent net change in the annual rate of SLCF emissions.

The coefficient *g* is a correction to *s* required to equate the radiative forcing of a SLCF directly to CO_2_ forcing without reference to the temperature response^38^. Specifically, *g* is a function of *s* $\left(g=\frac{(1-{e}^{(-s/(1-s))})}{s}\right)$ and holds a value of 1.13 for *s* = 0.25 (ref. ^38^). To calculate the response of GMST to emissions of the SLCF, ${E}_{C{O}_{2}-{e}_{100}}$ is substituted into Eq. (1) in place of ${E}_{C{O}_{2}}$.

IPCC AR6 explicitly notes that the GMST response to SLCFs such as CH_4_ is more accurately reproduced when using the GWP* approach (Eq. 3) than the GWP_100_ approach (Eq. 2)^6^. Treating CH_4_ as a long-lived gas (i.e. calculating its CO_2_ equivalent emissions using Eq. (2)) leads to overestimation of the GMST response to constant methane emissions over a multi-decadal period and a corresponding underestimation of the GMST response to any additional emissions that have been introduced over a more recent period (e.g. 20 years)^6,37,39^.

Here, we use Eq. (3) to estimate the cumulative CO_2_-equivalent emissions of CH_4_ $\left({E}_{C{O}_{2}-{e}_{100}}\right)$. The GWP_100_ values used in Eq. (3) were 29.8 for fossil CH_4_ emissions and 27.2 for LULUCF CH_4_ emissions, as reported in IPCC AR6 (difference between the two is the addition of CO_2_ to the atmosphere after fossil CH_4_ is oxidised)^6,39^. In addition, we use a time horizon of H = 100 years and a recent time period of Δ*t* = 20 years for assessment of the GMST response to net change in the annual rate of CH_4_ emissions. For the other constants in Eq. 3, we adopt the values published by Smith *et al*. (ref. ^38^), specifically: s = 0.25; g = 1.13. This combination of coefficient values is identical to that derived by Smith *et al*. (ref. ^38^) and employed in IPCC AR6^6^.

For the calculation of cumulative CO_2_-equivalent $\left({E}_{C{O}_{2}-{e}_{100}}\right)$ emissions of CH_4_ using Eq. (3) during years 1850–1869, an emissions time series for 1830–1849 is also required (noting that Δ*t* = 20 years). ${E}_{C{O}_{2}-{e}_{100}}$ was initially calculated for years 1850–2021 using the cumulative emissions of CH_4_ (*E*_*SL*_) since 1850 and net changes in the annual rate of SLCF emissions $\left(\frac{\Delta {E}_{SL\left(t-\Delta t\right)}}{\Delta t}\right)$ prior to 1850 are also included where necessary. Thereafter, the estimates of ${E}_{C{O}_{2}-{e}_{100}}$ for years 1851–2021 were re-based to the year 1850 by subtracting the value of ${E}_{C{O}_{2}-{e}_{100}}$ in year 1850 from its value in each year 1851–2021.

We substituted the resulting value of ${E}_{C{O}_{2}-{e}_{100}}$ into Eq. (1) in place of ${E}_{C{O}_{2}}$ to estimate the GMST response to emissions of CH_4_ during 1851–2021 (since the base year 1850). We adopted the TCRE value of 0.45 °C per 10^3^ Pg CO_2_ in Eq. (1) as discussed above (see section ‘**Global Mean Surface Temperature Response to Cumulative CO**_**2**_ **Emissions**’).

### National and international contributions to warming

The express Eqs. 1–3 were applied as described above to the global emissions records *and* to the records of emissions for individual countries and country groupings, *and* with subdivisions for fossil and LULUCF emissions. Thereafter, the fractional contributions of each country to change in GMST was estimated by dividing the GMST response to national emissions by the GMST response to global emissions. Note that the contributions of each sector (fossil and LULUCF) to change in GMST sum linearly to the national contributions to change in GMST, while the national contributions to change in GMST sum linearly to the global change in GMST. Hence the estimates of GMST response from our express approach are explicitly additive, allowing decomposition of warming contributions to countries and sectors.

The contributions of various country groupings to emissions and the GMST response to emissions were calculated by summing the contributions of constituent nations. The country groupings considered in this study were as follows. Several groupings derive from UNFCCC definitions (https://unfccc.int/parties-observers), including the 42 Annex I parties, the 23 Annex II parties (the most economically developed members of the Annex I), the 15 Economies in Transition (EIT; the lesser-developed members of Annex I), the 47 Least Developed Countries (LDCs), and the group of 24 Like-Minded Developing Countries (LMDC). In addition, we consider the contributions of the group of 38 countries of the Organisation for Economic Co-operation and Development (OECD; https://www.oecd.org/about/document/ratification-oecd-convention.htm), the group of 27 members of the European Union (EU27; https://european-union.europa.eu/principles-countries-history/country-profiles_en), and the Brazil, South Africa, India and China (BASIC) group. Lists of the countries included in each country grouping are provided in the **Data Records**^44^.

### Uncertainty assessment

Here, we characterise the uncertainties for all terms in Eqs. (1–3) at a consistent 1-sigma level (68% confidence interval), thus enabling the propagation of errors in related work if desired. We do not provide explicit estimates of uncertainty within our data records^44^.

#### Uncertainty in emissions estimates

Our estimates of CO_2_ emissions derive from the global carbon budget of the GCP^1,32,33^. The GCP provides an expert judgement of the uncertainty in its CO_2_ emissions estimates. At the global scale and in Annex I countries reporting to the UNFCCC, the GCP estimates that 1-sigma uncertainties in annual fossil CO_2_ emissions are 5%^3,40,64^. For non-Annex countries the GCP estimates a 10% uncertainty in annual fossil CO_2_ emissions due to less stringent reporting and verification. Meanwhile, the GCP estimates the 1-sigma uncertainty in global LULUCF CO_2_ emissions to be 50%^3^. The uncertainty on national scales is poorly constrained but likely higher than 50%^3^. We note that data relating to LULUCF emissions in China are subject to considerable uncertainty because the LUH2 and FRA datasets show opposing signs^51^. FRA exhibits large-scale forest plantation in China since the mid 20th Century, leading to an LULUCF sink, whereas LUH2 indicates widespread forest loss in China over the same period^51^.

As discussed above, Minx *et al*. (ref. ^64^) compared available estimates of CH_4_ and N_2_O emissions and found that PRIMAP-hist (TP scenario) lies centrally amongst those estimates. The spread of the estimates may be partly indicative of uncertainty in CH_4_ and N_2_O, although commonalities in parameter choice and requisite data sources amongst the emissions datasets means that uncertainties are likely to be larger than inferred by the spread of estimates alone^44^. Minx *et al*. (refs. ^58^,^64^) provide a best judgement of 1-sigma uncertainty in total CH_4_ emissions during 1970–2018 of ± 30% globally, with higher uncertainties nationally and for earlier decades^44^. Their current best judgement of 1-sigma uncertainty in total N_2_O emissions during 1970–2018 is ± 60% globally, and higher nationally and for earlier decades^64^. Hence, we note that the CH_4_ and N_2_O emissions estimates used in the current study lie centrally within a large uncertainty range globally and within a poorly constrained uncertainty range on national scales.

#### Uncertainty in the transient climate response to cumulative CO_2_ emissions

The IPCC AR6^2,6^ TCRE (*k* in Eq. (1)) considered 27 assessments of the TCRE value published between 2009 and 2021 and revised the earlier estimate provided in AR5 based on 17 subsequent estimates. Each estimate involved a different model or model ensemble exhibiting a particular climate sensitivity, with some studies constraining their estimates to observed changes in surface temperature. The revised best estimate for TCRE was 1.65 °C per 10^3^ Pg C emitted (0.45 °C per 10^3^ Pg CO_2_ emitted)^4,6^ with a 90% confidence interval of 1.0–2.3 °C per 10^3^ Pg C emitted, corresponding to 1-sigma uncertainty of 0.4 °C per 10^3^ Pg C emitted (0.18 °C per 10^3^ Pg CO_2_ emitted).

#### Uncertainty in the global warming potential of CH_4_ and N_2_O

The IPCC AR6^2^ reviewed the current understanding of uncertainty in the global warming potential of CH_4_ and N_2_O and many other anthropogenic gases and aerosols. Based on the latest evidence, the report arrives at a current best estimate of 29.8 for the GWP_100_ of fossil CH_4_, with an uncertainty of ± 11.9 at the 90% confidence interval, corresponding to a 1-sigma uncertainty of ± 7.1. The GWP_100_ of biogenic CH_4_ is reported to be 27.2 with an uncertainty of ± 10.9 at the 90% confidence interval, corresponding to 1-sigma uncertainty of ± 6.5. The major sources of uncertainty that contribute to the overall uncertainty in the GWP_100_ of CH_4_ are (in order of magnitude): (i) the absolute global warming potential of CO_2_ (the time-integrated radiative forcing caused by a CO_2_ emissions pulse over the 100-year horizon); (ii) the measurement of radiative efficiency of CH_4_ (absorption of energy across a spectrum of wavelengths); (iii) the atmospheric lifetime of the gas, and; (iv) chemistry feedbacks (interactions with other atmospheric gases). The GWP_100_ of N_2_O is reported to be 273 with an uncertainty of ± 128.3 at the 90% confidence interval, corresponding to 1-sigma uncertainty of ± 76.4. The major sources of uncertainty that contribute to the overall uncertainty in the GWP_100_ of N_2_O differ from those of CH_4_ and are (in order of magnitude): (i) chemistry feedbacks; (ii) the absolute global warming potential of CO_2_, and; (iii) the measurement of radiative efficiency of N_2_O.

#### Other uncertainties

The coefficient *s* relates to the rate of decline of radiative forcing resulting from the deep ocean thermal adjustment to recent forcing changes, *ρ* (see **Methods**). The value of *ρ* used here (0.33% year^−1^) reflects a multi-model average deep ocean adjustment period of around 300 years (1/*ρ*). The deep ocean adjustment period depends on the climate model used to derive it, with a 1-sigma range of around ± 110 years^36,76^. The uncertainty range of the deep ocean adjustment period infers an uncertainty in *ρ* of 0.24–0.51% year^−1^ at the 1-sigma interval. Finally, *g* in Eq. (3) is a function of *s* (see **Methods**) such that its uncertainty corresponds to that of *s*.

## Data Records

All records are available via a Zenodo data repository (ref. ^44^). The data records include three comma separated values (.csv) files as described below. All files are in ‘long’ format with one value provided in the *Data* column for each combination of the categorical variables *Year, Country Name, Country ISO3 code, Gas*, and *Component* columns. The *Component* field specifies fossil emissions, LULUCF emissions or total emissions of the gas. *Gas* specifies CO_2_, CH_4_, N_2_O or the three-gas total (labelled 3-GHG). ISO3 codes are specifically the unique ISO 3166-1 alpha-3 codes of each country (https://www.iso.org/iso-3166-country-codes.html).

### EMISSIONS_ANNUAL_1830–2021.csv (26.3 MB)

*Data* includes annual emissions of CO_2_ (Pg CO_2_ year^−1^), CH_4_ (Tg CH_4_ year^−1^) and N_2_O (Tg N_2_O year^−1^) during 1830–2021. The *Data* column provides values for every combination of the categorical variables. There are 369,048 data rows in the current version. Note that data for the years 1830–1849 are provided as these data are needed for the calculation of CO_2_-equivalent emissions of CH_4_ using the GWP* approach during years 1850–1869 (see Eq. 3).

### EMISSIONS_CUMULATIVE_CO2e100_1851–2021.csv (33.8 MB)

*Data* includes the cumulative CO_2_ equivalent emissions in units Pg CO_2_-e_100_ during 1851–2021. The *Data* column provides values for every combination of the categorical variables. There are 450,585 data rows in the current version.

### GMST_response_1851–2021.csv (28.9 MB)

*Data* includes the change in global mean surface temperature (GMST) due to emissions of the three gases during 1851–2021 in units °C. The *Data* column provides values for every combination of the categorical variables. There are 450,585 data rows in the current version.

In addition to the data records above, we provide lists of the countries included in each of the following country groupings in a Microsoft Excel workbook named **COUNTY_GROUPINGS**.**xlsx** (**~20 KB**): the 42 Annex I parties; the 23 Annex II parties; the 15 Economies in Transition (EIT); the 47 Least Developed Countries (LDCs); the 24 Like-Minded Developing Countries (LMDC); the 38 countries of the Organisation for Economic Co-operation and Development (OECD); the 27 members of the European Union (EU27), and the four countries of the BASIC group. Each group occupies one worksheet of the Excel workbook and consists of one column of listed countries.

## Technical Validation

We provide Figs. 1–8, Tables 1–4, and the text below to assist with the technical validation of our dataset including its cross-comparison with other studies.

**Fig. 1:**
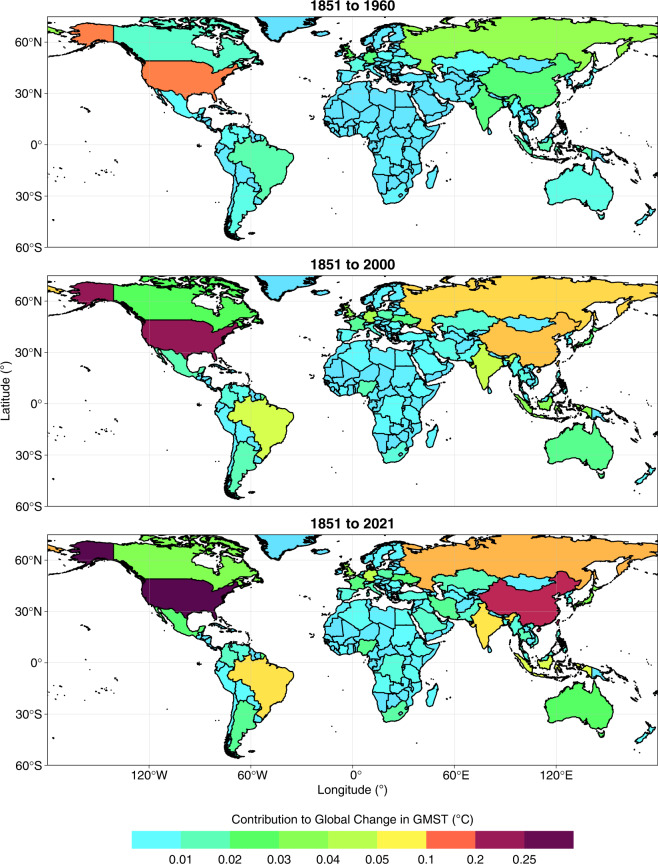
National contributions to change in global mean surface temperature (GMST, °C) resulting from historical emissions of CO_2_, CH_4_ and N_2_O during three time periods. All data shown are provided in the **Data Records**^44^.

**Fig. 2:**
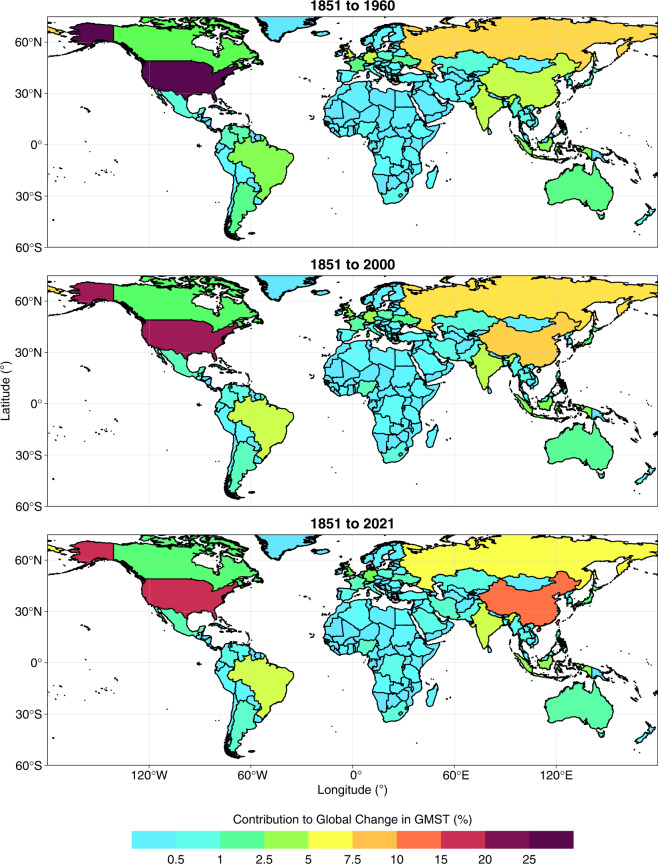
National contributions to change in global mean surface temperature (GMST) resulting from historical emissions of CO_2_, CH_4_ and N_2_O during three time periods. Values are expressed as a percentage of the change in GMST due to all global emissions of the three gases. All data shown are provided in the **Data Records**^44^.

**Fig. 3:**
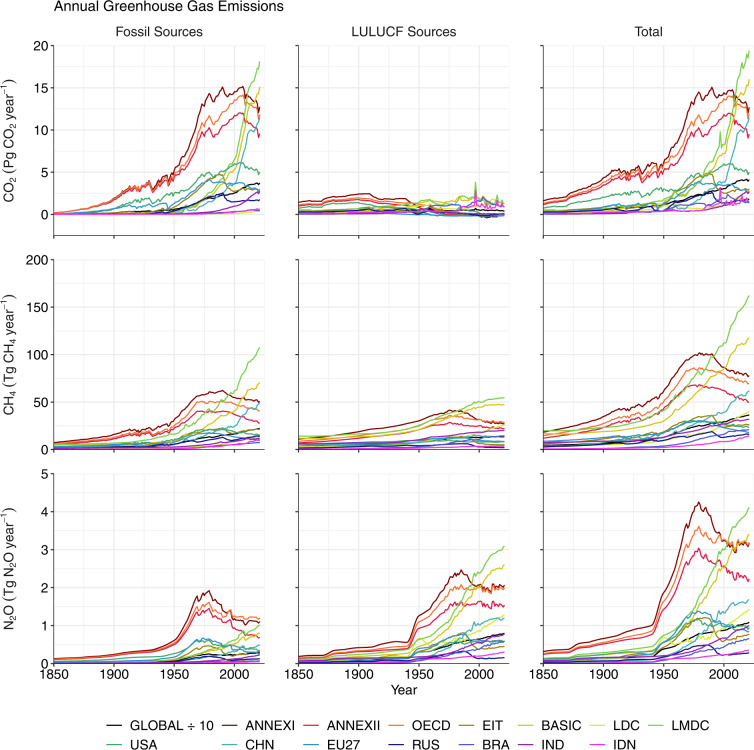
Annual emissions of CO_2_ (Pg CO_2_ year^−1^), CH_4_ (Tg CH_4_ year^−1^) and N_2_O (Tg N_2_O year^−1^) during 1850–2021, shown globally and for selected countries and country groupings. The primary data sources are the global carbon budget and PRIMAP-hist^3,40–42^ (see **Methods** for data sources). The ISO3 codes of countries in the legend are: CHN, China; RUS, Russia; BRA, Brazil; IND, India; IDN, Indonesia. All data shown are provided in the **Data Records**^44^.

**Fig. 4:**
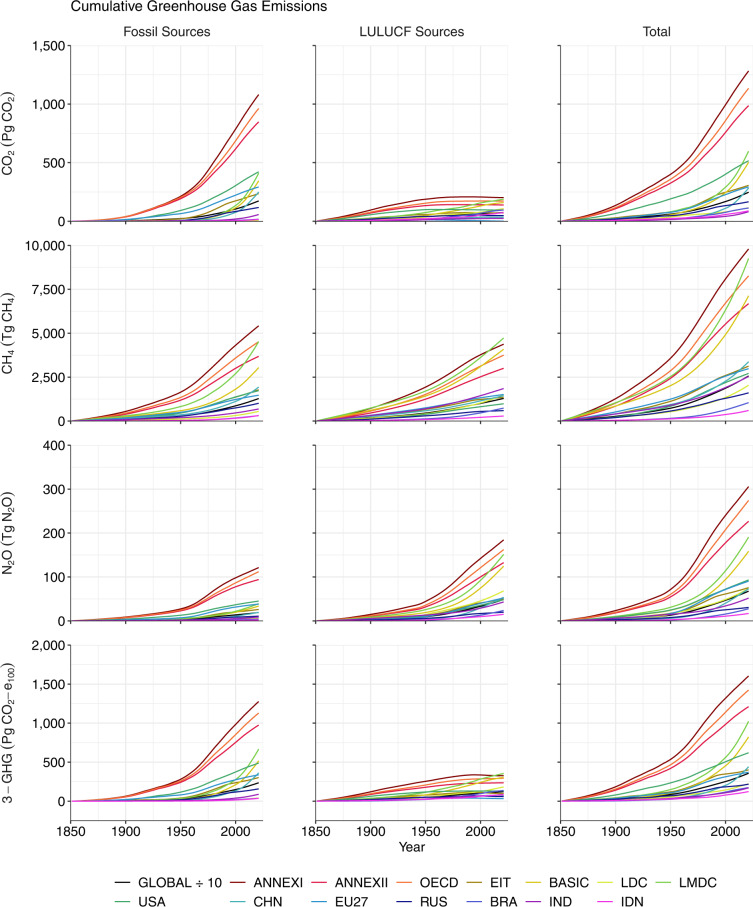
Cumulative emissions of CO_2_ (Pg CO_2_), CH_4_ (Tg CH_4_) and N_2_O (Tg N_2_O) and their sum (3-GHG; Pg CO_2_-e_100_) during 1851–2021, shown globally and for selected regions. Emissions of CH_4_ and N_2_O are converted to units Pg CO_2_-e_100_ using the GWP* approach^38^ and summed to a 3-GHG total (bottom row). The ISO3 codes of countries in the legend are: CHN, China; RUS, Russia; BRA, Brazil; IND, India; IDN, Indonesia. All data shown are provided in the **Data Records**^44^.

**Fig. 5:**
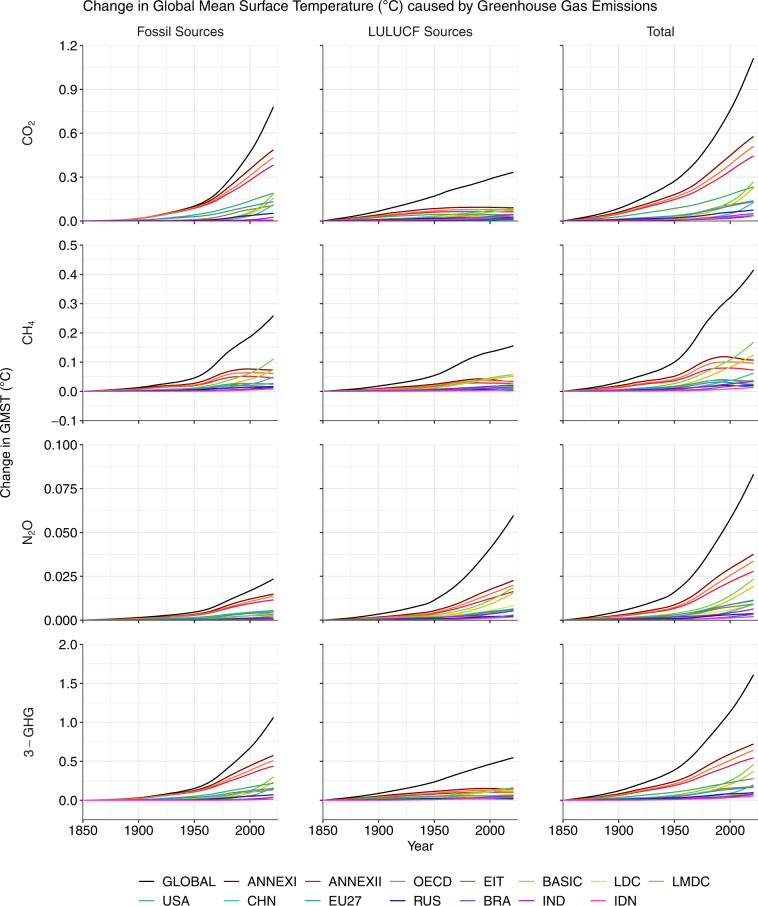
Response of global mean surface temperature (GMST, °C) to emissions of CO_2_, CH_4_ and N_2_O during 1851–2021, shown globally and for selected regions. The GMST response to historical CH_4_ emissions began to fall in some developed regions towards the end of the 20th Century due to a decline in CH_4_ emissions versus prior decades (see Fig. 3). The temperature response is calculated by multiplying the transient response to cumulative emissions of CO_2_ (TCRE) by the total emissions of CO_2_, CH_4_ and N_2_O expressed in Pg CO_2_-e_100_. Emissions of CH_4_ and N_2_O are converted to units Pg CO_2_-e_100_ using the GWP* approach^38^. The ISO3 codes of countries in the legend are: CHN, China; RUS, Russia; BRA, Brazil; IND, India; IDN, Indonesia. All data shown are provided in the **Data Records**^44^.

**Fig. 6:**
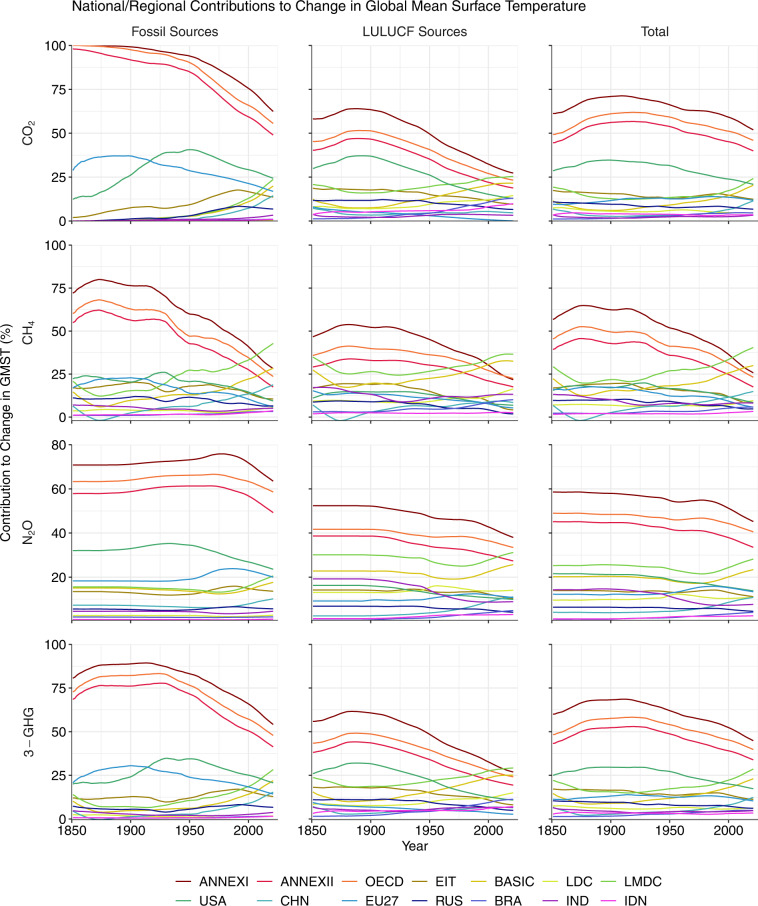
National or regional contributions (%) to change in global mean surface temperature (GMST) during 1851–2021, shown for selected regions. The ISO3 codes of countries in the legend are: CHN, China; RUS, Russia; BRA, Brazil; IND, India; IDN, Indonesia. All data shown are provided in the **Data Records**^44^.

**Fig. 7:**
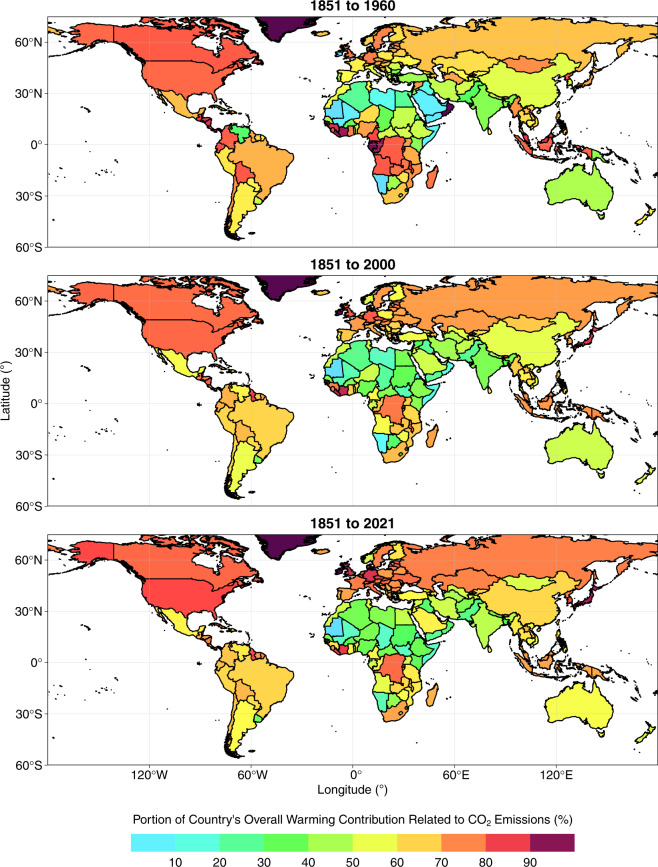
Percentage of each country’s total contribution to change in global mean surface temperature (GMST) related to emissions of CO_2_ (as opposed to CH_4_ or N_2_O), over three time periods. All data shown are provided in the **Data Records**^44^.

**Fig. 8:**
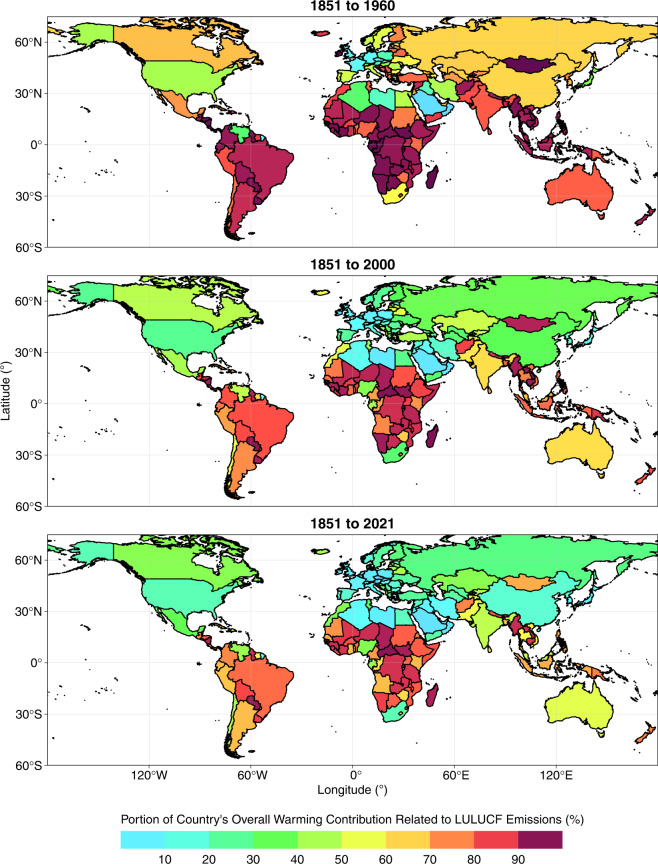
Percentage of each country’s total contribution to change in global mean surface temperature (GMST) related to emissions from land use, land use change and forestry (LULUCF; as opposed to fossil sources), over three time periods. These data relate to the total CO_2_-equivalent emissions of CO_2_, CH_4_ and N_2_O. All data shown are provided in the **Data Records**^44^.

**Table 1:** Change in global mean surface temperature (GMST) due to emissions of CO_2_ during 1851–2021, and the contributions of countries or country groupings which contributed at least 3% of the change in GMST.

Gas	Source	Country or Grouping	°C	%
CO_2_	Total	GLOBAL	1.11	
		ANNEXI	0.58	51.9
		OECD	0.51	45.9
		NONANNEX	0.51	45.8
		ANNEXII	0.44	40.0
		LMDC	0.27	24.2
		**USA**	**0**.**23**	**20**.**9**
		BASIC	0.23	20.4
		EIT	0.14	12.4
		***EU27***	***0****.****13***	***11****.****8***
		**China**	**0**.**13**	**11**.**5**
		**Russia**	**0**.**07**	**6**.**7**
		LDC	0.05	4.6
		**Brazil**	**0**.**05**	**4**.**6**
		**Germany**	**0**.**04**	**3**.**7**
		**Indonesia**	**0**.**04**	**3**.**6**
		**India**	**0**.**04**	**3**.**3**
		**United Kingdom**	**0**.**03**	**3**.**1**
	Fossil	GLOBAL	0.78	
		ANNEXI	0.49	62.4
		OECD	0.43	55.6
		ANNEXII	0.38	49.0
		NONANNEX	0.27	34.3
		**USA**	**0**.**19**	**24**.**4**
		LMDC	0.18	23.7
		BASIC	0.16	19.9
		***EU27***	***0****.****13***	***16****.****9***
		**China**	**0**.**11**	**14**.**4**
		EIT	0.11	13.6
		**Russia**	**0**.**05**	**6**.**8**
		**Germany**	**0**.**04**	**5**.**4**
		**United Kingdom**	**0**.**03**	**4**.**3**
		**Japan**	**0**.**03**	**3**.**9**
		**India**	**0**.**03**	**3**.**3**
	LULUC	GLOBAL	0.33	
		NONANNEX	0.24	72.6
		ANNEXI	0.09	27.3
		LMDC	0.08	25.4
		OECD	0.08	23.3
		BASIC	0.07	21.5
		ANNEXII	0.06	18.8
		LDC	0.05	14.5
		**Brazil**	**0**.**04**	**13**.**0**
		**USA**	**0**.**04**	**12**.**8**
		**Indonesia**	**0**.**03**	**9**.**9**
		EIT	0.03	9.6
		**Russia**	**0**.**02**	**6**.**5**
		**China**	**0**.**02**	**4**.**6**
		**Canada**	**0**.**01**	**3**.**4**
		**India**	**0**.**01**	**3**.**2**

Bold text is used to highlight individual countries as opposed to country groupings, while bold italicised text is used to highlight the European Union as its 27 constituent countries share common environmental, agricultural and climate policies. The **Data Records**^44^ include all data shown, as well as the values for all countries and country groupings.

**Table 2:** Change in global mean surface temperature (GMST) due to emissions of CH_4_ during 1851–2021, and the contributions of countries or country groupings which contributed at least 3% of the change in GMST.

Gas	Source	Country or Grouping	°C	%
CH_4_	Total	GLOBAL	0.41	
		NONANNEX	0.31	74.0
		LMDC	0.17	40.5
		BASIC	0.12	29.9
		ANNEXI	0.11	25.8
		OECD	0.10	23.2
		ANNEXII	0.07	17.5
		**China**	**0**.**06**	**14**.**9**
		LDC	0.04	9.6
		**USA**	**0**.**04**	**8**.**5**
		**India**	**0**.**03**	**8**.**2**
		EIT	0.03	8.1
		**Brazil**	**0**.**02**	**5**.**9**
		***EU27***	***0****.****02***	***5****.****8***
		**Russia**	**0**.**02**	**4**.**8**
		**Indonesia**	**0**.**01**	**3**.**3**
	Fossil	GLOBAL	0.26	
		NONANNEX	0.18	71.5
		LMDC	0.11	42.8
		BASIC	0.07	28.4
		ANNEXI	0.07	28.2
		OECD	0.06	23.6
		**China**	**0**.**05**	**18**.**8**
		ANNEXII	0.05	17.5
		EIT	0.03	10.5
		**USA**	**0**.**02**	**9**.**4**
		**Russia**	**0**.**02**	**6**.**5**
		***EU27***	***0****.****02***	***6****.****0***
		LDC	0.01	5.5
		**India**	**0**.**01**	**5**.**1**
		**Indonesia**	**0**.**01**	**3**.**7**
		**Nigeria**	**0**.**01**	**3**.**7**
		**Brazil**	**0**.**01**	**3**.**3**
	LULUC	GLOBAL	0.16	
		NONANNEX	0.12	78.2
		LMDC	0.06	36.6
		BASIC	0.05	32.5
		OECD	0.04	22.5
		ANNEXI	0.03	21.7
		ANNEXII	0.03	17.6
		LDC	0.03	16.4
		**India**	**0**.**02**	**13**.**3**
		**Brazil**	**0**.**02**	**10**.**1**
		**China**	**0**.**01**	**8**.**5**
		USA	0.01	6.9
		*EU27*	*0.01*	*5.3*
		EIT	0.01	4.2
		**Pakistan**	**0**.**00**	**3**.**2**
		**Australia**	**0**.**00**	**3**.**1**

Bold text is used to highlight individual countries as opposed to country groupings, while bold italicised text is used to highlight the European Union as its 27 constituent countries share common environmental, agricultural and climate policies. The **Data Records**^44^ include all data shown, as well as the values for all countries and country groupings.

**Table 3:** Change in global mean surface temperature (GMST) due to emissions of N_2_O during 1851–2021, and the contributions of countries or country groupings which contributed at least 3% of the change in GMST.

Gas	Source	Country or Grouping	°C	%
N_2_O	Total	GLOBAL	0.083	
		NONANNEX	0.045	54.7
		ANNEXI	0.038	45.2
		OECD	0.034	40.5
		ANNEXII	0.028	33.5
		LMDC	0.023	28.2
		BASIC	0.019	23.4
		**USA**	**0**.**011**	**13**.**8**
		***EU27***	***0****.****011***	***13****.****4***
		EIT	0.009	11.2
		LDC	0.009	10.9
		**China**	**0**.**009**	**10**.**8**
		**India**	**0**.**006**	**7**.**7**
		**Russia**	**0**.**004**	**4**.**5**
		**Brazil**	**0**.**003**	**4**.**1**
		**Australia**	**0**.**003**	**3**.**8**
	Fossil	GLOBAL	0.024	
		ANNEXI	0.015	63.5
		OECD	0.014	58.6
		ANNEXII	0.012	49.2
		NONANNEX	0.009	36.2
		**USA**	**0**.**006**	**23**.**6**
		LMDC	0.005	20.6
		*EU27*	*0.005*	*20.0*
		BASIC	0.004	17.7
		EIT	0.003	13.6
		**China**	**0**.**002**	**10**.**2**
		**Russia**	**0**.**001**	**5**.**6**
		**India**	**0**.**001**	**4**.**6**
		**Germany**	**0**.**001**	**4**.**5**
		**Mexico**	**0**.**001**	**3**.**6**
		**France**	**0**.**001**	**3**.**6**
		**United Kingdom**	**0**.**001**	**3**.**5**
	LULUC	GLOBAL	0.060	
		NONANNEX	0.037	61.9
		ANNEXI	0.023	38.0
		OECD	0.020	33.4
		LMDC	0.019	31.2
		ANNEXII	0.016	27.3
		BASIC	0.015	25.7
		LDC	0.008	14.1
		**China**	**0**.**007**	**11**.**0**
		***EU27***	***0****.****006***	***10****.****8***
		EIT	0.006	10.2
		**USA**	**0**.**006**	**9**.**9**
		**India**	**0**.**005**	**8**.**9**
		**Brazil**	**0**.**003**	**4**.**9**
		**Australia**	**0**.**003**	**4**.**9**
		**Russia**	**0**.**002**	**4**.**1**
		**Indonesia**	**0**.**002**	**3**.**1**

Bold text is used to highlight individual countries as opposed to country groupings, while bold italicised text is used to highlight the European Union as its 27 constituent countries share common environmental, agricultural and climate policies. The **Data Records**^44^ include all data shown, as well as the values for all countries and country groupings.

**Table 4:** Change in global mean surface temperature (GMST) due to combined emissions of CO_2_, CH_4_ and N_2_O during 1851–2021, and the contributions of countries or country groupings which contributed at least 3% of the change in GMST.

Gas	Source	Country or Grouping	°C	%
3-GHG	Total	GLOBAL	1.61	
		NONANNEX	0.86	53.5
		ANNEXI	0.72	44.8
		OECD	0.64	39.8
		ANNEXII	0.55	33.8
		LMDC	0.46	28.6
		BASIC	0.37	23.0
		**USA**	**0**.**28**	**17**.**3**
		**China**	**0**.**20**	**12**.**3**
		EIT	0.18	11.2
		***EU27***	***0****.****17***	***10****.****4***
		LDC	0.10	6.2
		**Russia**	**0**.**10**	**6**.**1**
		**Brazil**	**0**.**08**	**4**.**9**
		**India**	**0**.**08**	**4**.**8**
		**Indonesia**	**0**.**06**	**3**.**4**
	Fossil	GLOBAL	1.06	
		ANNEXI	0.57	54.1
		OECD	0.51	47.9
		NONANNEX	0.46	43.4
		ANNEXII	0.44	41.3
		LMDC	0.30	28.3
		BASIC	0.23	21.9
		**USA**	**0**.**22**	**20**.**7**
		**China**	**0**.**16**	**15**.**4**
		***EU27***	***0****.****15***	***14****.****3***
		EIT	0.14	12.8
		**Russia**	**0**.**07**	**6**.**7**
		**Germany**	**0**.**05**	**4**.**3**
		**India**	**0**.**04**	**3**.**8**
		**United Kingdom**	**0**.**04**	**3**.**4**
	LULUC	GLOBAL	0.55	
		NONANNEX	0.40	73.0
		LMDC	0.16	29.2
		ANNEXI	0.15	26.9
		BASIC	0.14	25.1
		OECD	0.13	24.2
		ANNEXII	0.11	19.4
		LDC	0.08	15.0
		**Brazil**	**0**.**06**	**11**.**3**
		**USA**	**0**.**06**	**10**.**8**
		EIT	0.04	8.1
		**Indonesia**	**0**.**04**	**7**.**1**
		**India**	**0**.**04**	**6**.**7**
		**China**	**0**.**04**	**6**.**4**
		**Russia**	**0**.**03**	**4**.**9**

Bold text is used to highlight individual countries as opposed to country groupings, while bold italicised text is used to highlight the European Union as its 27 constituent countries share common environmental, agricultural and climate policies. The **Data Records**^44^ include all data shown, as well as the values for all countries and country groupings.

### Cumulative emissions

According to our estimates, 2,471 Pg CO_2_ were emitted globally during 1851–2021 (Figs. 3, 4) leading to a CO_2_-induced increase in GMST of 1.11 °C (Fig. 5, Table 1). We estimate that cumulative fossil CO_2_ emissions during 1851–2021 amounted to 1,732 Pg CO_2_ (Figs. 3, 4) and caused warming of 0.78 °C (Fig. 5, Table 1). The dataset of fossil CO_2_ emissions is taken directly from the GCP^40^ such that the cumulative emissions for 1851–2021 match that reported in the GCB (ref. ^3^). Our estimate of cumulative LULUCF CO_2_ emissions during 1851–2021 is 739 Pg CO_2_ (Figs. 3, 4) leading to warming of 0.33 °C (Fig. 5, Table 1). The estimate of global cumulative LULUCF CO_2_ emissions also matches the value reported in the GCB^3^ because the GCB also uses the average estimate from the three products employed here^49–51^. Thus, our estimates of total annual and cumulative CO_2_ emissions match the estimates made by the GCP on the global scale^3^.

According to the PRIMAP-hist HISTTP dataset, 921 Pg CO_2_-e_100_ of CH_4_ (25,669 Tg CH_4_) were emitted globally during 1851–2021 (Figs. 3, 4) leading to a 0.41 °C increase in GMST (Fig. 5, Table 2). We estimate that cumulative fossil CH_4_ emissions during 1851–2021 amounted to 575 Pg CO_2_-e_100_ (12,803 Tg CH_4,_ Figs. 3, 4) and caused warming of 0.26 °C (Fig. 5, Table 2), while cumulative LULUCF CH_4_ emissions amounted to 347 Pg CO_2_-e_100_ (12,866 Tg CH_4_, Figs. 3, 4) and caused warming of 0.16 °C (Fig. 5, Table 2). Also relevant to the calculation of CO_2_-equivalent emission of CH_4_ are the net changes in the rate of annual emissions over the past 20 years ($\frac{\Delta {E}_{SL\left(t-\Delta t\right)}}{\Delta t}$ in Eq. (3)), which were 334 Tg CH_4_ year^−1^ during 2002–2021 including 198 Tg CH_4_ year^−1^ from fossil sources and 135 Tg CH_4_ year^−1^ from LULUCF sources (Fig. 3). Our CH_4_ emissions estimates are taken directly from the PRIMAP-hist dataset such that the cumulative emissions for 1851–2021 match the PRIMAP-hist record^41,42^.

According to the PRIMAP-hist HISTTP dataset, 185 Pg CO_2_−e_100_ of N_2_O (677 Tg N_2_O, Figs. 3, 4) were emitted globally during 1851–2021 leading to a 0.08 °C increase in GMST (Fig. 5, Table 3). We estimate that cumulative fossil N_2_O emissions during 1851–2021 amounted to 52 Pg CO_2_−e_100_ (191 Tg N_2_O) and caused warming of 0.02 °C (Fig. 5, Table 3), while cumulative LULUCF N_2_O emissions amounted to 133 Pg CO_2_−e_100_ (485 Tg N_2_O, Figs. 3, 4) and caused warming of 0.06 °C (Fig. 5, Table 3). Our N_2_O emissions estimates are taken directly from the PRIMAP-hist dataset such that the cumulative emissions for 1851–2021 match the PRIMAP-hist record^41,42^.

Minx *et al*. (ref. ^64^) recently compared seven estimates of total CH_4_ and N_2_O emissions for the years 1970–2018 and found that the PRIMAP-hist dataset (with the HISTTP scenario used here) yields estimates that lie centrally within the range of available estimates. For example, the PRIMAP-hist estimate for total fossil CH_4_ emissions in 2010 is around 340 Tg CH_4_ year^−1^, and there are three higher estimates (up to 360 Tg CH_4_ year^−1^) and three lower estimates (lowest estimate 300 Tg CH_4_ year^−1^). Also for the year 2010, the PRIMAP-hist estimate for total fossil N_2_O emissions in 2010 is around 9.5 Tg N_2_O year^−1^, and there are two higher estimates (up to 12 Tg N_2_O year^−1^) and four lower estimates (lowest estimate 8.5 Tg N_2_O year^−1^). PRIMAP-hist similarly lies centrally amongst estimates of CH_4_ and N_2_O emissions in the years 1970, 1980, 1990 and 2018 (see Fig. 1c,d of ref. ^64^).

### Change in global mean surface temperature

We compared our estimates of change in GMST (Fig. 5) caused by historical emissions of CO_2_, CH_4_, and N_2_O with the values reported recently by the IPCC in AR6^1,8^. The IPCC AR6 estimated the change in GMST caused by CO_2_, CH_4_ and N_2_O emissions to be 1.4°C between 1850-1900 and 2010-2019^1^,^8^. For a close comparison, we calculated the change in GMST between the periods 1851-1900 and 2010-2019 to be 1.39 °C in our dataset.

The IPCC AR6 estimated the change in GMST caused by cumulative CO_2_ emissions from all sources to be 0.8 °C between 1850–1900 and 2010–2019, with an uncertainty range of 0.5–1.2 °C (90% confidence interval)^1,8^. For a close comparison, we calculated the change in GMST between the periods 1851–1900 and 2010–2019 from our dataset to be 0.96°C. Hence, our estimate of warming caused by global historical CO_2_ emissions lies within the very likely range identified by the IPCC, and 0.16 °C above the IPCC’s central estimate.

The IPCC AR6 estimated the change in GMST due to historical CH_4_ emissions from all sources to be 0.5 °C between 1850–1900 and 2010–2019, with an uncertainty range of 0.3–0.8 °C (90% confidence interval)^1,8^. For a close comparison, we calculated the change in GMST between 1851–1900 and 2010–2019 to be 0.37 °C in our dataset. Hence, our estimate of warming caused by global historical CH_4_ emissions lies within the very likely range identified by the IPCC, and 0.13 °C below the central estimate.

The IPCC AR6 estimated the change in GMST due to historical N_2_O emissions from all sources to be 0.09 °C between 1850–1900 and 2010–2019, with an uncertainty range of 0.05–0.16 °C (90% confidence interval)^1,8^. For a close comparison, we calculated the change in GMST between 1851–1900 and 2010–2019 to be 0.07 °C in our dataset. Hence, our estimate of warming caused by global historical N_2_O emissions lies within the very likely range identified by the IPCC, and 0.02 °C below the central estimate.

### National contributions to warming

#### Contributions through CO_2_ emissions

The ranking of countries by national contributions to warming through CO_2_ emissions presented here compares well with those published previously. For example, the list of top-10 contributors to the warming caused by total CO_2_ emissions during 1851–2021 (USA, China, Russia, Brazil, Germany, Indonesia, India, UK, Japan and Canada; Table 1, Figs. 5, 6) shares many similarities with the list presented by Matthews *et al*. (ref. ^12^ USA, China, Russia, Brazil, India, Germany, UK, France, Indonesia and Canada) for the years 1800–2005. Skeie *et al*.^12^ also arrived at a similar list (USA, EU28, China, Russia, Indonesia, Brazil, Japan, India, Canada) for the years 1850–2012 (note that the EU27 post-Brexit also ranks between the USA and China in our dataset; Table 1, Figs. 5, 6). Some differences in rank between studies may be explained by differences in the period of emissions considered. For example, our estimates indicate that China moved into second position ahead of Russia since 2005 based on its evolving emissions since 1850, while India rose from tenth position to fifth position ahead of Indonesia, Germany, the UK, Japan and Canada. Other differences versus previous work may result from revisions to emissions estimates, and we particularly note that the order of national contributions to warming caused by LULUCF CO_2_ emissions shared fewer similarities with that of previous work than in the case of warming related to fossil CO_2_ emissions. This broadly aligns with expectations as LULUCF CO_2_ emissions estimates carry greater uncertainty and are subject to more frequent and substantial revisions than fossil CO_2_ emissions estimates^3,77^.

Overall contributions to warming through total CO_2_ emissions mask large differences in the relative contributions of fossil and LULUCF emissions at national levels (Table 1, Fig. 8). For example, Brazil’s contribution to warming from LULUCF CO_2_ emissions (0.04 °C, Table 1, Figs. 5, 6) is greater than any other nation’s contribution and accounts for most of its overall contribution to warming through total CO_2_ emissions (0.05 °C, Table 1, Figs. 5, 6). Indonesia’s contributions to warming through historical CO_2_ emissions are also dominated by LULUCF emissions. These observations are in line with prior reports of a strong contribution of LULUCF emissions to total CO_2_ emissions in Brazil and Indonesia^78^. These examples highlight how excluding LULUCF emissions can lead to a substantial underestimation of the contribution of some nations to warming^12,13,16^. In stark contrast, LULUCF emissions during 1851–2021 were negative in several European countries (including Germany and France) and thus the LULUCF sector in these countries contributed to a cooling of GMST, slightly offsetting the warming associated with their fossil CO_2_ emissions. The cooling effect results from negative cumulative emissions from the LULUCF sector of these countries since 1850 as observed by Friedlingstein *et al*. (ref. ^3^). This pattern may in part reflect unaccounted LULUCF emissions of CO_2_ that occurred as a result of land use changes in Europe prior to 1850, although previous work has established that accounting for preindustrial LULUCF prior to 1850 impacts the share a region takes of global warming only by a few percent and is thus of similar magnitude as uncertainties related to other methodological choices (for the region itself, however, its contribution may be altered substantially in relative terms)^16,79^.

Globally, 30% of the warming caused by total CO_2_ emissions during 1851–2021 is associated with LULUCF, but with largely varying shares in different countries (Fig. 8). The contribution of LULUCF CO_2_-related warming to total CO_2_-related warming lies around the global value in Russia and India, whereas the LULUCF share of CO_2_-related warming is lower in the US (18%) and China (12%) and higher in Brazil (85%) and Indonesia (83%). The cooling related to LULUCF CO_2_ emissions in Germany and France offsets 3–6% of the warming related to their fossil CO_2_ emissions. While the developed members of the OECD and Annex II contribute more towards fossil CO_2_-related warming than the like-minded developing countries (LMDC) and non-Annex groups, the opposite is true with respect to LULUCF CO_2_-related warming.

Our focus here has been on contributions to warming during the period 1851–2021, however we note that national contributions have evolved through time since 1850 (Figs. 5, 6). For example, the contributions of the LMDC group to LULUCF CO_2_-related only overtook the contributions of the OECD around the year 2011.

#### Effect of including methane and nitrous oxide

Major shares of global CH_4_ and N_2_O emissions are associated with land use (Tables 2, 3; Figs. 3, 4)^4,5^. The GCP has estimated that livestock (enteric fermentation and manure management) and rice cultivation contributed almost 40% of global total CH_4_ emission during 2009–2018^4^, and that nitrogen fertilizer use, livestock and manure management contribute 50% of global total N_2_O emissions during 2007–2016^5^. Consequently, previous studies have found that considering emissions of CH_4_ and N_2_O tends to increase the contributions to warming of countries with high agricultural intensity and area under management^12,13^. We observe similar patterns in our dataset (Tables 2, 3; Figs. 5–8). Globally, the LULUCF sector accounted for 38% of the total warming from CH_4_ emissions and 72% of the warming from N_2_O emissions during 1851–2021 (Tables 2, 3; Figs. 5, 6). Notably, a considerably greater fraction of the warming caused by CH_4_ emissions was associated with LULUCF in some countries (e.g. over 60% in India, Brazil, Australia and Pakistan; Table 2; Figs. 5, 6). When considering CH_4_ and N_2_O emissions, the contribution to warming of India, China, and Brazil rose by 110%, 56% and 55%, respectively, relative to the CO_2_-related warming alone (Tables 1, 4; Figs. 5–7). For comparison, the additional contribution to warming of most other large emitters (USA, EU27, Russia, Canada) was 30% or lower, and values were below 15% in Germany, the UK and Japan.

The differential effects of including CH_4_ and N_2_O in assessments of national contributions to warming leads to a re-ordering of the top contributors (Table 4) as compared to the scenario in which only CO_2_-related warming is considered (Table 1; Figs. 5, 6). For example, India moves from 7^th^ to 5^th^ position above Indonesia and Germany, and China’s contribution also moves beyond that of the EU27. Hence we highlight the critical importance of study design when ranking the contributions of individual nations to warming in line with previous studies^12,13^. Moreover, we note that emissions of CH_4_ and N_2_O are more uncertain than emissions of fossil CO_2_ and so any changes in the ranking of contributors related to the additional consideration of CH_4_ and N_2_O should be treated with particular scrutiny.

Due to re-equilibration of atmospheric CH_4_ concentrations within a short period after a change in annual emissions, it is possible for a reduction in the rate of annual CH_4_ emissions to bring about a cooling effect on GMST even if the annual emissions remain positive, unlike in the case of CO_2_ of N_2_O emissions. For an example of this effect, see Figs. 5, 6 where China’s contribution to change in GMST is negative (signifying a cooling effect) during years 1866–1885. China’s mean annual CH_4_ emissions fell by around 8% between the 1850s and 1870s according to the PRIMAP-hist dataset (Fig. 3), resulting in negative cumulative CO_2_-equivalent emissions in line with Eq. (3) and a small cooling effect on GMST until the year 1885 (Figs. 5, 6). Similarly, emissions of CH_4_ began to decline in many developed nations in the late decades of the 20th Century, resulting in a reduced contribution of these countries to warming since the mid-20^th^ Century (Figs. 5, 6). In contrast, cooling can only result from a reduction in CO_2_ and N_2_O emissions if cumulative emissions become negative, owing to the linear relationship between their cumulative emissions and change in GMST (Eqs. 1, 2).

## Usage Notes

In addition to the uncertainties evaluated in the **Methods**, we highlight some aspects of our study design which might affect our assessment of national contributions to warming versus alternative methodologies.

### Limitations of TCRE and GWP*

While use of TCRE and GWP* to attribute historical warming has the advantages of simplicity, transparency and alignment with the latest IPCC assessment, this approach also has limitations stemming from the fact that TCRE and GWP* are first-order approximations of a complex dynamic system in which radiatively active species directly or indirectly interact. TCRE is a linear approximation of the long-term global temperature response to an emission of CO_2_ that is assumed to be constant through time and independent of the emission intensity or past emissions. However, individual models typically exhibit a more complex dynamic response^74^, as also illustrated by the existence of a committed warming after reaching net zero CO_2_ emissions (the Zero-Emissions Commitment)^80^, and by the breaking of the linear approximation in case of significant temperature overshoot^81^. In addition, TCRE is used indiscriminately for both fossil and LULUCF sources, whereas LULUCF CO_2_ emissions are known not to be precisely equivalent to fossil ones^82^ because they are mostly caused by land cover change that simultaneously reduces the land carbon sink^83^, thereby changing the TCRE itself. In the GCB^3^, this is termed the loss of additional sink capacity^3,51,84^, and accounting for it would slightly increase the relative contribution of countries having emitted significant amounts of LULUCF CO_2_. Also, LULUCF exerts an associated biophysical effect on climate via changes in planetary surface albedo that is not paralleled in activities leading to fossil CO_2_ emission. The biophysical effect causes differences in the climatic response to LULUCF versus fossil CO_2_ emission, however this distinction is not accounted for in the TCRE value^85^.

GWP* is a variation of the classic GWP that enables definition of CO_2_-equivalence within the TCRE framework. It remedies what is perhaps the most critical shortcoming of GWP when applied to short-lived species: a lack of explicit time dynamic. The GWP* dynamic remains simplistic, however, compared to what would be obtained with non-linear models with a detailed evolution of a species’ atmospheric lifetimes^86^. Furthermore, GWP* uses GWP in its formulation, and therefore includes only the atmo- and biogeo-chemical feedbacks accounted for within the chosen GWP value. For instance, our chosen GWP for CH_4_ includes effects on tropospheric ozone and the carbon cycle through the climate feedback^2,87^, and it has implicit backgrounds of atmospheric CH_4_ and emission of ozone precursors^2^. In GWP and GWP*, these factors and feedbacks are linearised, assumed constant in time, and attributed to the main species of interest, which essentially ignores the complex real-life cross-species dynamics^2,88^. Accounting for these, however, requires more advanced models^89–91^ that come at the cost of the simplicity and transparency we were aiming at, for changes in national contributions that would likely be of second-order.

### Other considerations related to study design

Many foregoing studies have highlighted the importance of perspective when assigning contributions to climate change, specifically referring to the role of study design in determining contributions^13,16,18,21,22,28,92–97^. Our results similarly point to various structural elements of study design that influence the assessed contributions to warming, which we summarise here.

First, contributions to warming depend on the gases and aerosols considered in the analysis. Different anthropogenic activities emit various gases and aerosols at ranging intensities (e.g. industrial versus agricultural). Each country has a unique environmental and socioeconomic situation causing differences in the prevalence of source activities and influencing emission rates of associated gases and aerosols. Consequently, a country’s contribution to warming increases if a gas or aerosol associated with one of its prevalent activities is considered in the assessment. For example, the inclusion of CH_4_ and N_2_O enhances the contribution to warming of countries with intensive or extensive agriculture^12,13^. Here, we consider only CO_2_, CH_4_ and N_2_O emissions in our assessment of national contributions to warming, thus excluding national contributions to warming through emissions of other radiatively active species. The IPCC AR6 finds that anthropogenic emissions of black carbon aerosols, halogenated gases (CFC + HCFC + HFC) and volatile organic compounds and carbon monoxide (NMVOC + CO) cause a warming at the global scale comparable to that of N_2_O^1,8^. Inclusion of these species thus has potential to influence national contributions to warming to a similar degree as the inclusion of N_2_O. Note that N_2_O-related warming contributes around 7% of the warming related to all three GHGs in this analysis on average across individual countries (standard deviation 5%). In addition, the cooling effect of sulphate aerosols and other reflective aerosol species is not included here, yet we note that the cooling effect of aerosols is comparable in magnitude to the warming effect of CH_4_ at the global scale^1,8^. Consequently, changes in national contributions to warming would occur if other gases and aerosols were to be included in this analysis. For example, including aerosols has been estimated to reduce China’s contribution to warming to 8%, as compared with 11% in a case including only well-mixed GHGs^24^.

Second, contributions to warming depend on the time period under consideration. For example, the inclusion or exclusion of pre-industrial LULUCF CO_2_ emissions has a small influence on the contribution made by countries whose key period of land use change preceded the industrial period (up to a few percentage points in European countries and China)^16^. Here, we consult multi-gas emissions datasets that collectively include the years 1851–2021, and we report on contributions to climate change since 1850 (note that the CH_4_ emissions data for years 1830–1849 are also required to calculate cumulative CO_2_-equivalent emissions from 1850 onwards, see **Methods**). Figures 5, 6 show how national contributions to warming have evolved with time since 1850. However, we note that earlier or later reference years would provide a different perspective on national contributions to emissions. For example, selecting a reference year of 1900 would reduce cumulative global CO_2_ emissions by 40 Pg CO_2_ and lessen the related warming by 0.02 °C (−2.3%). For national contributions, the corresponding effect of varying the reference year on warming would depend on the fraction of cumulative national emissions that occurred before or after the reference year for any particular country. A change in reference year within 1850–1900 has a considerably smaller impact on the GMST responses to global or national CH_4_ emissions due to lesser dependence of CH_4_-related warming on cumulative emissions than in the case of CO_2_ or N_2_O.

Third, contributions to warming depend on population. We do not include per capita emissions or per capita contributions to warming in our dataset. Nonetheless, we note that previous work has highlighted per capita expressions of emissions or warming as a means of accounting for differences in the intensity of emissions or warming impact per country, providing further perspective on the national accountability for climate change^3,12^.

Finally, contributions to warming depend on international trade. Some countries (e.g. China and India, and Brazil) emit CO_2_ in the process of producing goods or services for export (in *net* terms), while other countries/regions (e.g. the EU27 and the USA) are *net* importers and consume goods or services which require emissions in external territories. Here, we do not account for national emissions embodied in goods or services traded between countries (i.e. the emissions estimates used here include territorial emissions only rather than consumption-based emissions). Estimates of consumption-based emissions are available for fossil CO_2_^33^ and for LULUCF CO_2_, CH_4_ and N_2_O^98^ and could be used to produce consumption-based national warming contributions, however these records begin only in the 1960s–1970s.

## Data Availability

The R Statistics code used to perform all methods described here can be accessed via the GitHub repo at the following link: https://github.com/jonesmattw/National_Warming_Contributions.git.

## References

[1] IPCC. Summary for Policymakers. In: *Climate Change 2021: The Physical Science Basis. Contribution of Working Group I to the Sixth Assessment Report of the Intergovernmental Panel on Climate Change* [Masson-Delmotte, V. *et al* (eds.)]. Cambridge University Press, Cambridge, United Kingdom and New York, NY, USA, pp. 3−32, 10.1017/9781009157896.001 (2021).

[2] Forster, P. *et al*. The Earth’s Energy Budget, Climate Feedbacks, and Climate Sensitivity. In: *Climate Change 2021: The Physical Science Basis. Contribution of Working Group I to the Sixth Assessment Report of the Intergovernmental Panel on Climate Change* [Masson-Delmotte, V. *et al* (eds.)] Cambridge University Press, Cambridge, United Kingdom and New York, NY, USA, pp. 923–1054, 10.1017/9781009157896.009 (2021).

[3] Friedlingstein P (2022). Global Carbon Budget 2022. Earth Syst. Sci. Data.

[4] Saunois M (2020). The Global Methane Budget 2000–2017. Earth Syst. Sci. Data.

[5] Tian H (2020). A comprehensive quantification of global nitrous oxide sources and sinks. Nature.

[6] Canadell, J. G. *et al*. Global Carbon and other Biogeochemical Cycles and Feedbacks. In: *Climate Change 2021: The Physical Science Basis. Contribution of Working Group I to the Sixth Assessment Report of the Intergovernmental Panel on Climate Change* [Masson-Delmotte, V. *et al* (eds.)]. Cambridge University Press, Cambridge, United Kingdom and New York, NY, USA, pp. 673–816, 10.1017/9781009157896.007 (2021).

[7] Gulev, S. K. *et al*. Changing State of the Climate System. In: *Climate Change 2021: The Physical Science Basis. Contribution of Working Group I to the Sixth Assessment Report of the Intergovernmental Panel on Climate Change* [Masson-Delmotte, V. *et al* (eds.)]. Cambridge University Press, Cambridge, United Kingdom and New York, NY, USA, pp. 287–422, 10.1017/9781009157896.004 (2021).

[8] IPCC WGI. Intergovernmental Panel on Climate Change Working Group 1: Summary for Policymakers of the Working Group I Contribution to the IPCC Sixth Assessment Report - data for Figure SPM.2 (v20210809) [CEDA Archive], available at: https://data.ceda.ac.uk/badc/ar6_wg1/data/spm/spm_02/v20210809, last access: 23rd January 2023 (2021).

[9] UNFCCC. United Nations Framework Convention on Climate Change, Nationally determined contributions under the Paris Agreement. Synthesis report by the secretariat, available at: https://unfccc.int/ndc-synthesis-report-2022, last access: 23rd January 2023. (2022).

[10] Le Quéré C (2021). Fossil CO2 emissions in the post-COVID-19 era. Nat. Clim. Change.

[11] Le Quéré C (2019). Drivers of declining CO2 emissions in 18 developed economies. Nat. Clim. Change.

[12] Matthews HD (2014). National contributions to observed global warming. Environ. Res. Lett..

[13] Skeie RB (2017). Perspective has a strong effect on the calculation of historical contributions to global warming. Environ. Res. Lett..

[14] Friedlingstein P, Solomon S (2005). Contributions of past and present human generations to committed warming caused by carbon dioxide. Proc. Natl. Acad. Sci..

[15] Wei T (2012). Developed and developing world responsibilities for historical climate change and CO2 mitigation. Proc. Natl. Acad. Sci..

[16] Pongratz J, Caldeira K (2012). Attribution of atmospheric CO 2 and temperature increases to regions: importance of preindustrial land use change. Environ. Res. Lett..

[17] Lewis SC, Perkins-Kirkpatrick SE, Althor G, King AD, Kemp L (2019). Assessing Contributions of Major Emitters’ Paris-Era Decisions to Future Temperature Extremes. Geophys. Res. Lett..

[18] Den Elzen M, Schaeffer M (2002). Responsibility for Past and Future Global Warming: Uncertainties in Attributing Anthropogenic Climate Change. Clim. Change.

[19] Höhne N (2011). Contributions of individual countries’ emissions to climate change and their uncertainty. Clim. Change.

[20] Ekwurzel B (2017). The rise in global atmospheric CO2, surface temperature, and sea level from emissions traced to major carbon producers. Clim. Change.

[21] den Elzen M (2005). Analysing countries’ contribution to climate change: scientific and policy-related choices. Environ. Sci. Policy.

[22] den Elzen MGJ, Olivier JGJ, Höhne N, Janssens-Maenhout G (2013). Countries’ contributions to climate change: effect of accounting for all greenhouse gases, recent trends, basic needs and technological progress. Clim. Change.

[23] Ward DS, Mahowald NM (2014). Contributions of developed and developing countries to global climate forcing and surface temperature change. Environ. Res. Lett..

[24] Li B (2016). The contribution of China’s emissions to global climate forcing. Nature.

[25] Skeie RB, Peters GP, Fuglestvedt J, Andrew R (2021). A future perspective of historical contributions to climate change. Clim. Change.

[26] Fu B (2021). The contributions of individual countries and regions to the global radiative forcing. Proc. Natl. Acad. Sci..

[27] Fu B (2022). Climate Warming Mitigation from Nationally Determined Contributions. Adv. Atmospheric. Sci..

[28] Otto FEL, Skeie RB, Fuglestvedt JS, Berntsen T, Allen MR (2017). Assigning historic responsibility for extreme weather events. Nat. Clim. Change.

[29] Callahan CW, Mankin JS (2022). National attribution of historical climate damages. Clim. Change.

[30] Williams RG, Ceppi P, Katavouta A (2020). Controls of the transient climate response to emissions by physical feedbacks, heat uptake and carbon cycling. Environ. Res. Lett..

[31] Allen MR (2009). Warming caused by cumulative carbon emissions towards the trillionth tonne. Nature.

[32] Gillett NP, Arora VK, Matthews D, Allen MR (2013). Constraining the Ratio of Global Warming to Cumulative CO2 Emissions Using CMIP5 Simulations. J. Clim..

[33] Millar RJ, Friedlingstein P (2018). The utility of the historical record for assessing the transient climate response to cumulative emissions. Philos. Trans. R. Soc. Math. Phys. Eng. Sci..

[34] Arora VK (2020). Carbon–concentration and carbon–climate feedbacks in CMIP6 models and their comparison to CMIP5 models. Biogeosciences.

[35] Allen MR (2018). A solution to the misrepresentations of CO2-equivalent emissions of short-lived climate pollutants under ambitious mitigation. Npj Clim. Atmospheric Sci..

[36] Cain M (2019). Improved calculation of warming-equivalent emissions for short-lived climate pollutants. Npj Clim. Atmospheric Sci..

[37] Lynch J, Cain M, Pierrehumbert R, Allen M (2020). Demonstrating GWP\ast: a means of reporting warming-equivalent emissions that captures the contrasting impacts of short- and long-lived climate pollutants. Environ. Res. Lett..

[38] Smith MA, Cain M, Allen MR (2021). Further improvement of warming-equivalent emissions calculation. Npj Clim. Atmospheric Sci..

[39] Allen MR (2022). Indicate separate contributions of long-lived and short-lived greenhouse gases in emission targets. Npj Clim. Atmospheric Sci..

[40] Andrew RM, Peters GP (2022). Zenodo.

[41] Gütschow J (2016). The PRIMAP-hist national historical emissions time series. Earth Syst. Sci. Data.

[42] Gütschow J, Pflüger M (2022). Zenodo.

[43] Hong C (2021). Global and regional drivers of land-use emissions in 1961–2017. Nature.

[44] Jones MW (2023). Zenodo.

[45] IPCC. Technical Summary. In: Climate Change 2021: The Physical Science Basis. Contribution of Working Group I to the Sixth Assessment Report of the Intergovernmental Panel on Climate Change [Masson-Delmotte, V. *et al* (eds.)]. Cambridge University Press, Cambridge, United Kingdom and New York, NY, USA, pp. 33−144, 10.1017/9781009157896.002 (2021).

[46] Gilfillan, D. & Marland, G. CDIAC-FF: global and national CO_2_ emissions from fossil fuel combustion and cement manufacture: 1751–2017. Earth Syst. *Sci. Data* **13**, 1667–1680 (2021).

[47] BP. BP: Statistical Review of World Energy 2022, available at: https://www.bp.com/en/global/corporate/energy-economics/statistical-review-of-world-energy.html, last access: 23rd January 2023 (2023).

[48] Andrew, R. M. Global CO_2_ emissions from cement production, 1928–2018. Earth Syst. *Sci. Data* **11**, 1675–1710 (2019).

[49] Hansis E, Davis SJ, Pongratz J (2015). Relevance of methodological choices for accounting of land use change carbon fluxes. Glob. Biogeochem. Cycles.

[50] Houghton RA, Nassikas AA (2017). Global and regional fluxes of carbon from land use and land cover change 1850–2015: Carbon Emissions From Land Use. Glob. Biogeochem. Cycles.

[51] Gasser T (2020). Historical CO2 emissions from land use and land cover change and their uncertainty. Biogeosciences.

[52] van der Werf GR (2017). Global fire emissions estimates during 1997–2016. Earth Syst. Sci. Data.

[53] Hooijer A (2010). Current and future CO2 emissions from drained peatlands in Southeast Asia. Biogeosciences.

[54] Qiu C (2021). Large historical carbon emissions from cultivated northern peatlands. Sci. Adv..

[55] Conchedda G, Tubiello FN (2020). Drainage of organic soils and GHG emissions: validation with country data. Earth Syst. Sci. Data.

[56] Hurtt GC (2020). Harmonization of global land use change and management for the period 850–2100 (LUH2) for CMIP6. Geosci. Model Dev..

[57] FAO. Global Forest Resources Assessment 2020: Main report. 10.4060/ca9825en (FAO, 2020).

[58] FAOSTAT. FAOSTAT: Food and Agriculture Organization Statistics Division, Statistical Database, domains Climate Change, available at: https://www.fao.org/faostat/en/#data/GT, last access: 23rd January 2023) (2021).

[59] Klein Goldewijk K, Beusen A, Doelman J, Stehfest E (2017). Anthropogenic land use estimates for the Holocene – HYDE 3.2. Earth Syst. Sci. Data.

[60] UNFCCC. United Nations Framework Convention on Climate Change, National Inventory Submissions, available at: https://unfccc.int/ghg-inventories-annex-i-parties/2022, last access: 23rd January 2023 (2022).

[61] Crippa M (2020). High resolution temporal profiles in the Emissions Database for Global Atmospheric Research. Sci. Data.

[62] Janssens-Maenhout G (2019). EDGAR v4.3.2 Global Atlas of the three major greenhouse gas emissions for the period 1970–2012. Earth Syst. Sci. Data.

[63] Hoesly RM (2018). Historical (1750–2014) anthropogenic emissions of reactive gases and aerosols from the Community Emissions Data System (CEDS). Geosci. Model Dev..

[64] Minx JC (2021). A comprehensive and synthetic dataset for global, regional, and national greenhouse gas emissions by sector 1970–2018 with an extension to 2019. Earth Syst. Sci. Data.

[65] Millar RJ (2017). Emission budgets and pathways consistent with limiting warming to 1.5 °C. Nat. Geosci..

[66] Rogelj J, Forster PM, Kriegler E, Smith CJ, Séférian R (2019). Estimating and tracking the remaining carbon budget for stringent climate targets. Nature.

[67] Jones CD, Friedlingstein P (2020). Quantifying process-level uncertainty contributions to TCRE and carbon budgets for meeting Paris Agreement climate targets. Environ. Res. Lett..

[68] Matthews HD (2021). An integrated approach to quantifying uncertainties in the remaining carbon budget. Commun. Earth Environ..

[69] UNEP. United Nations Environment Programme - Copenhagen Climate Centre (UNEP-CCC): The Emissions Gap Report 2022, available at: https://www.unep.org/resources/emissions-gap-report-2022, last access: 23rd January 2023 (2022).

[70] Etminan, M., Myhre, G., Highwood, E. J. & Shine, K. P. Radiative forcing of carbon dioxide, methane, and nitrous oxide: A significant revision of the methane radiative forcing. *Geophys. Res. Lett*. **43** (2016).

[71] Hodnebrog Ø (2020). Updated Global Warming Potentials and Radiative Efficiencies of Halocarbons and Other Weak Atmospheric Absorbers. Rev. Geophys..

[72] Denison S, Forster PM, Smith CJ (2019). Guidance on emissions metrics for nationally determined contributions under the Paris Agreement. Environ. Res. Lett..

[73] Smith SM (2012). Equivalence of greenhouse-gas emissions for peak temperature limits. Nat. Clim. Change.

[74] Joos F (2013). Carbon dioxide and climate impulse response functions for the computation of greenhouse gas metrics: a multi-model analysis. Atmospheric Chem. Phys..

[75] Myhre, G. *et al*. Anthropogenic and Natural Radiative Forcing. In: Climate Change 2013: The Physical Science Basis. Contribution of Working Group I to the Fifth Assessment Report of the Intergovernmental Panel on Climate Change [Stocker, T.F., D. Qin, G.-K. Plattner, M. Tignor, S.K. Allen, J. Boschung, A. Nauels, Y. Xia, V. Bex and P.M. Midgley (eds.)]. Cambridge University Press, Cambridge, United Kingdom and New York, NY, USA (2013).

[76] Geoffroy O (2013). Transient Climate Response in a Two-Layer Energy-Balance Model. Part I: Analytical Solution and Parameter Calibration Using CMIP5 AOGCM Experiments. J. Clim..

[77] Bastos A (2021). Comparison of uncertainties in land-use change fluxes from bookkeeping model parameterisation. Earth Syst. Dyn..

[78] Crippa M (2021). Food systems are responsible for a third of global anthropogenic GHG emissions. Nat. Food.

[79] Pongratz, J., Raddatz, T., Reick, C. H., Esch, M. & Claussen, M. Radiative forcing from anthropogenic land cover change since A.D. 800. *Geophys. Res. Lett*. **36** (2009).

[80] MacDougall AH (2020). Is there warming in the pipeline? A multi-model analysis of the Zero Emissions Commitment from CO_2_. Biogeosciences.

[81] Gasser T (2018). Path-dependent reductions in CO2 emission budgets caused by permafrost carbon release. Nat. Geosci..

[82] Gitz, V. & Ciais, P. Amplifying effects of land-use change on future atmospheric CO2 levels. *Glob. Biogeochem. Cycles* **17** (2003).

[83] Gasser T, Ciais P (2013). A theoretical framework for the net land-to-atmosphere CO2 flux and its implications in the definition of emissions from land-use change. Earth Syst. Dyn..

[84] Pongratz J, Reick CH, Houghton RA, House JI (2014). Terminology as a key uncertainty in net land use and land cover change carbon flux estimates. Earth Syst. Dyn..

[85] Simmons, C. T. & Matthews, H. D. Assessing the implications of human land-use change for the transient climateresponse to cumulative carbon emissions. *Environ. Res. Lett*. **11**, 035001 (2016).

[86] Prather, M. J., Holmes, C. D. & Hsu, J. Reactive greenhouse gas scenarios: Systematic exploration of uncertainties and the role of atmospheric chemistry. *Geophys. Res. Lett*. **39** (2012).

[87] Gasser T (2017). Accounting for the climate-carbon feedback in emission metrics. Earth Syst. Dyn..

[88] Fu B (2020). Short-lived climate forcers have long-term climate impacts via the carbon–climate feedback. Nat. Clim. Change.

[89] Leach NJ (2021). FaIRv2.0.0: a generalized impulse response model for climate uncertainty and future scenario exploration. Geosci. Model Dev..

[90] Gasser T (2017). The compact Earth system model OSCAR v2.2: description and first results. Geosci. Model Dev..

[91] Meinshausen M, Raper SCB, Wigley TML (2011). Emulating coupled atmosphere-ocean and carbon cycle models with a simpler model, MAGICC6 – Part 1: Model description and calibration. Atmospheric. Chem. Phys..

[92] Müller B, Höhne N, Ellermann C (2009). Differentiating (historic) responsibilities for climate change. Clim. Policy.

[93] Höhne N, den Elzen M, Escalante D (2014). Regional GHG reduction targets based on effort sharing: a comparison of studies. Clim. Policy.

[94] Frumhoff PC, Heede R, Oreskes N (2015). The climate responsibilities of industrial carbon producers. Clim. Change.

[95] Gignac R, Matthews HD (2015). Allocating a 2 °C cumulative carbon budget to countries. Environ. Res. Lett..

[96] Steininger KW, Lininger C, Meyer LH, Muñoz P, Schinko T (2016). Multiple carbon accounting to support just and effective climate policies. Nat. Clim. Change.

[97] Ciais P (2013). Attributing the increase in atmospheric CO2 to emitters and absorbers. Nat. Clim. Change.

[98] Hong C (2022). Land-use emissions embodied in international trade. Science.

